# Effects of transcranial alternating current stimulation to the supplementary motor area on motor learning

**DOI:** 10.3389/fnbeh.2024.1378059

**Published:** 2024-04-29

**Authors:** Shunpei Yamamoto, Shota Miyaguchi, Takuma Ogawa, Yasuto Inukai, Naofumi Otsuru, Hideaki Onishi

**Affiliations:** ^1^Graduate School, Niigata University of Health and Welfare, Niigata, Japan; ^2^Institute for Human Movement and Medical Sciences, Niigata University of Health and Welfare, Niigata, Japan

**Keywords:** transcranial alternating current stimulation, supplementary motor area, γ-oscillatory activity, visuomotor tracking task, motor learning

## Abstract

Transcranial alternating current stimulation (tACS) is a noninvasive method for brain stimulation that artificially modulates oscillatory brain activity in the cortical region directly beneath the electrodes by applying a weak alternating current. Beta (β) oscillatory activity in the supplementary motor area (SMA) is involved in motor planning and maintenance, whereas gamma (γ) oscillatory activity is involved in the updating of motor plans. However, the effect of applying tACS to the SMA on motor learning has not yet been investigated. This study assessed the effects of applying tACS to the SMA on motor learning. Forty-two right-handed healthy adults (age 20.6 ± 0.5 years, 24 men and 18 women) were included. Motor learning was assessed using a visuomotor tracking task with pinch tension of the right thumb and right forefinger. Each trial lasted 60 s, and the error rates were measured. Conductive rubber electrodes were attached to the SMA and the left shoulder for tACS. Stimulation was applied at an intensity of 1.0 mA and frequencies of 70 and 20 Hz in the γ-tACS and β-tACS treatment groups, respectively. The sham group was only administered a fade-in/out. The visuomotor tracking task was performed for 10 trials before tACS and 10 trials after tACS. Two trials were conducted on the following day to determine motor skill retention. The average deviation measured during 60 s was considered the error value. Pre-stimulation learning rate was calculated as the change in error rate. Post-stimulation learning rate and retention rate were calculated as the change in error rate after stimulation and on the day after stimulation, respectively. In both the stimulation groups, differences in pre-stimulation learning, post-stimulation learning, and retention rates were not significant. However, in the γ-tACS group, baseline performance and pre-stimulation learning rate were positively correlated with post-stimulation learning rate. Therefore, applying γ-tACS to the SMA can increase post-stimulation learning rate in participants exhibiting low baseline performance and high pre-stimulation learning rate. Our findings suggest that motor learning can be effectively enhanced by applying γ-tACS to the SMA based on an individual’s motor and learning abilities.

## 1 Introduction

Motor learning involves acquisition of motor skills. In motor learning, skills are acquired relatively quickly in the early stages and then improve slowly over multiple practice sessions in the later stages (Doyon and Benali, 2005; Dayan and Cohen, 2011). The late stage of learning is essential for long-term use of motor skills. We believe that motor skills can be further improved by enhancing learning efficiency during this period. Acquisition of motor skills during motor learning involves activities in various regions of the brain, including the primary motor cortex (M1), cerebellum, prefrontal cortex, premotor cortex, and supplementary motor area (SMA) (Jenkins et al., 1994; Karni et al., 1995). The SMA is a higher motor cortical area in Brodmann area 6 and is involved in regulating motor planning based on feedback information (Kasess et al., 2008), motor preparation and initiation, motor learning, and execution of complex motor tasks (Gross et al., 2005). SMA neurons are involved in updating and maintaining continuous motor planning and are activated before movement execution (Mushiake et al., 1991). Thus, the SMA formulates motor plans in advance of actual movement, serving as a crucial cortical region for executing movement. A study of oscillatory brain activity in the SMA demonstrated that β-oscillatory activity is involved in maintaining motor planning and γ-oscillatory activity is involved in updating motor plans (Hosaka et al., 2016). Therefore, β-and γ-oscillatory activities of the SMA play an important role in motor planning. We hypothesize that increasing γ-oscillatory activity, rather than β-oscillatory activity, during the latter half of learning (when skill improvement is slow) can promote motor learning by updating motor plans (Hosaka et al., 2016).

Transcranial alternating current stimulation (tACS) is a noninvasive brain stimulation method that modulates brain oscillations in cortical areas (Antal and Herrmann, 2016). The application of tACS through two electrodes attached to a participant’s head modulates brain oscillations to a specific frequency immediately below the electrodes (Helfrich et al., 2014a,b). This phenomenon is called entrainment, wherein endogenous oscillatory activity approximates externally applied frequency, and it is considered one of the mechanisms of tACS (Reato et al., 2013; Helfrich et al., 2014b; Lakatos et al., 2019; Vogeti et al., 2022). Previous studies have demonstrated that tACS modulates oscillatory brain activity directly beneath the electrode during and after stimulation (Neuling et al., 2013; Helfrich et al., 2014a,b), and the neurophysiological effects of tACS were observed for up to 30 min after stimulation (Herrmann et al., 2013). In recent years, the effects of tACS on motor learning have been investigated. A previous study reported that γ-band tACS (γ-tACS) administered to M1 improved performance when a typing task was repeated after stimulation, indicating that γ-tACS improves motor learning ability (Sugata et al., 2018). Administration of γ-tACS to M1 improved the performance of a finger visuomotor tracking task, and simultaneous administration of γ-tACS to M1 and the cerebellum improved retention of motor ability 1 day after exercise training (Santarnecchi et al., 2017; Miyaguchi et al., 2020b). Thus, application of tACS to M1 and the cerebellum may enhance motor learning and motor skills. However, studies on administering tACS to the SMA are limited. Many studies on tACS and motor learning have focused on the early stages of learning and the next-day retention stage, but few studies have focused on the later stages of learning.

We previously investigated the effects of applying tACS to the SMA on bimanual motor task performance (Miyaguchi et al., 2020a). In participants exhibiting lower baseline performance, γ-tACS improved motor performance; however, in those exhibiting higher baseline performance, β-tACS improved motor performance. These results suggest that the effectiveness of different stimulus frequencies depends on an individual’s motor ability (Miyaguchi et al., 2020a). However, this previous study only examined the immediate effects of tACS on motor skills and did not assess motor learning. Thus, the effect of applying tACS to the SMA on motor learning remains unclear. In another study, administration of γ-tACS to M1 enhanced motor learning capacity, whereas β-tACS did not have a similar effect (Sugata et al., 2018). As mentioned above, the β-oscillatory activity of the SMA is involved in maintaining motor plans, whereas the γ-oscillatory activity is involved in updating these plans (Hosaka et al., 2016). Based on this, when tACS is applied to the SMA after learning has progressed to a certain extent, β-tACS does not show significant changes, as evidenced in previous studies, but γ-tACS may lead to improved motor learning efficiency (Sugata et al., 2018). In the present study, we hypothesized that administering γ-tACS to the SMA would promote the updating of motor plans and improve motor learning efficiency after stimulation. We also established a β-tACS group to confirm whether this effect was specific to γ-tACS. We hypothesized that there would be no change in learning efficiency after stimulation in the β-tACS group due to maintenance of the motor plan. Therefore, this study aimed to determine the effect of applying tACS to the SMA on motor learning. Our findings may provide useful information for developing motor learning programs that contribute to motor planning.

## 2 Materials and methods

### 2.1 Participants

The study included 42 right-handed healthy adults without neurological disorders (age 20.6 ± 0.5 years, 24 men and 18 women). The participants had no metal in their bodies; no heart problems, such as arrhythmia; and were not taking any medication during the study. The participants were categorized into three groups (*n* = 14 per group): γ-tACS (age 20.6 ± 0.5 years, 8 men and 6 women); β-tACS (age 20.6 ± 0.5 years, 8 men and 6 women); and sham (age 20.6 ± 0.5 years, 8 men and 6 women). Handedness was evaluated using the Edinburgh handedness test (γ-tACS group: 94.1% ± 7.7%; β-tACS group: 94.1% ± 9.7%; and sham group: 94.8% ± 7.9%). There were no significant differences in age and handedness scores among the groups, and they were nearly equivalent (age: *p* = 1.000; handedness: *p* = 0.960). The study was conducted according to the principles of the Declaration of Helsinki and received approval from the Ethics Committee of Niigata University of Health and Welfare (approval number: 19224-240227). We explained the contents of the study to all participants and obtained their consent to participate.

### 2.2 tACS

tACS was performed using a constant-current transcranial stimulator (Nurostym tESr, NeuroDevice) and two conductive rubber electrodes (5 cm × 5 cm, 25 cm^2^) covered with a sponge saturated with physiological saline (tES Electrodes, Product number: NSEL35001). The electrodes and sponge were designed for use with the transcranial electrical stimulation device. The sponge covering the electrodes was soaked in physiological saline, and then a conductive gel was applied to the head-contact surface, reducing resistance and discomfort. The electrode attachment sites were in the SMA region (3.0 cm anterior to Cz based on the international 10–20 system) and on the left shoulder (Green et al., 2018; Manji et al., 2018; Miyaguchi et al., 2018). Following insights from earlier studies, we set the stimulation intensity at 1.0 mA (peak-to-peak), incorporated fade-in and fade-out periods lasting 5 s each, and designated stimulation frequencies of 70 and 20 Hz for the γ-tACS and β-tACS groups, respectively (Moisa et al., 2016; Miyaguchi et al., 2018). Stimulation time was set to 15 min based on previous studies demonstrating that tACS promotes motor learning (Bologna et al., 2019). In the sham group, the stimulus frequency was set at either 70 Hz or 20 Hz, and only a 10 s fade-in/out was performed. During stimulation, the participants rested in a seated position.

### 2.3 Visuomotor tracking task

**Figure 1:**
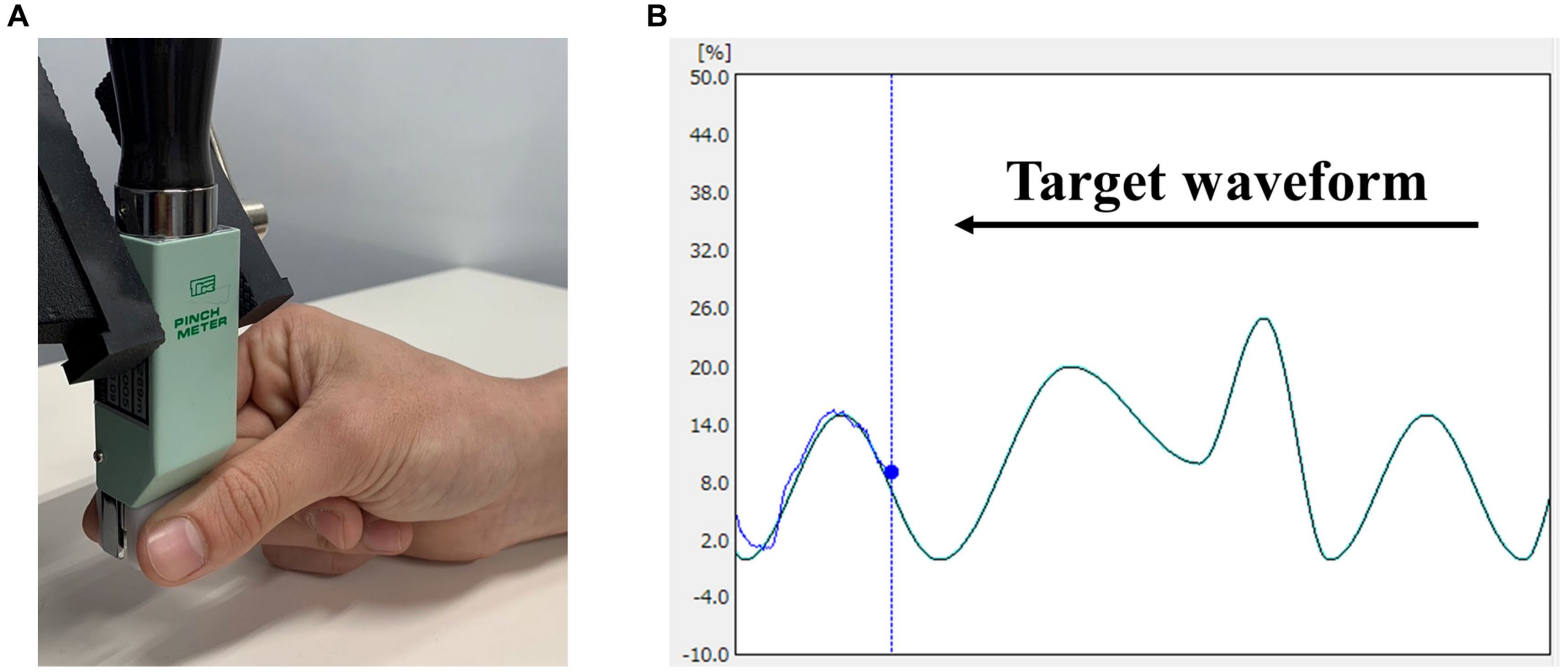
Visuomotor tracking task. **(A)** Pinch movements in the visuomotor tracking task. Pinch force between the participant’s right thumb and index finger was measured. **(B)** An example of the markers and waveforms used in the visuomotor tracking task. The light blue curve represents the target waveform, and the blue circle represents the target marker. Pinch tension was adjusted to accurately align the marker, which moved up and down in response to the pinch tension exerted by the participant, whereas waveforms flowed from right to left.

Visuomotor tracking tasks have been commonly employed in motor learning studies. Therefore, we assessed motor learning using a visuomotor tracking task, referencing our previous study (Miyaguchi et al., 2020b) (Figure 1). Motor task parameters were measured using a pinch tension meter (Takei Scientific Instruments, T.K.K. 1269n, Niigata, Japan) and force control software (Takei Scientific Instruments, Niigata, Japan). The participants were seated on a chair with their right elbow joint flexed, the forearm in an intermediate position, the wrist slightly dorsiflexed, and the forearm resting on the armrest. The pinch tension meter was operated by the participant using the right thumb and index finger. Pinch force was adjusted to accurately align the marker, which moved up and down in response to pinch force exerted by the participant, whereas the target waveform flowed from right to left on the laptop screen. Five variations of waveform times were included in the test: 1,111, 1,428, 1,666, 2,500, and 3,333 ms. Movement intensity was set in the range of 0–25% of the participant’s maximum pinch force, with five variations (0–10%, 0–15%, 5–15%, 0–20%, and 10–25%). This range was established to enable moderate learning without imposing excessive burden on the participants. Five combinations of movement intensity and waveform duration were selected to create a movement pattern (pattern A: 0–10%, 1,111 ms; pattern B: 0–15%, 2,500 ms; pattern C: 5–15%, 1,428 ms; pattern D: 0–20%, 3,333 ms; and pattern E: 10–25%, 1,666 ms). Each trial lasted 60 s and included the presentation of the five movement patterns six times in random order. Participants were instructed to trace the target waveform as accurately as possible. During each trial, the force control software was used to measure the deviation in pinch tension required to match the provided target waveform. The sampling frequency of the software was set to 100 Hz, and the amount of deviation during the trial, recorded to the third decimal place, was saved in an Excel file and used for analysis. The average of the deviations measured during 60 s was taken as the error value, and this value was normalized using each participant’s maximum tension to calculate the error rate for each trial.

### 2.4 Experimental procedure

**Figure 2:**
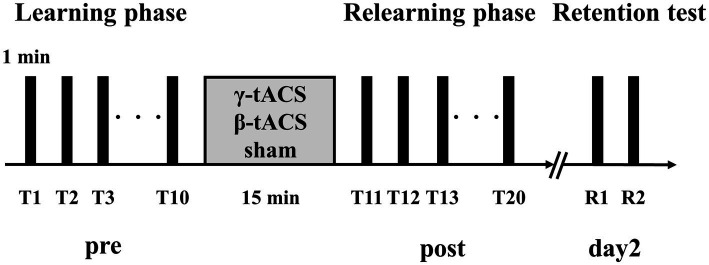
Experimental procedure. Participants performed 10 motor learning trials using a visuomotor tracking task before stimulation (pre). tACS was performed with the participant in a resting state. Then, 10 motor learning trials were conducted again (post). Two trials were performed 24 h after the second trial set (day 2).

Figure 2 shows the experimental procedure, which was based on previous studies on tACS and motor learning (Sugata et al., 2018). First, we measured maximum pinch force of the participant and set exercise intensity for the visuomotor tracking task. Next, the position of the SMA was identified based on the international 10–20 system (3 cm anterior to Cz). For the learning phase, we conducted 10 trials of the visuomotor tracking task (T1–T10). After the learning phase, tACS was applied for 15 min. Then, the visuomotor tracking task was repeated for 10 trials (T11–T20) for the relearning phase. The rest period between each trial was 60 s. Two trials (R1 and R2) were conducted 24 h later to determine the retention of motor skills. Additionally, we calculated the learning rate before tACS intervention (pre-stimulation learning rate), learning rate after tACS intervention (post-stimulation learning rate), and next-day retention rate (retention rate). The calculation method is explained in section 2.6.

### 2.5 Questionnaires

To examine side effects (phosphene, itching, etc.) during tACS and no stimulation, participants rated the intensity of side effects on a 7-point scale (0 = “no sensation” to 6 = “very strong sensation”) during tACS (Raco et al., 2014). They were asked about side effects at the beginning of stimulation (0 min), during stimulation (7.5 min), and after stimulation (15 min).

### 2.6 Data and statistical analysis

Pre-stimulation learning, post-stimulation learning, and retention rates were calculated using error rates obtained from visuomotor tracking tasks for each participant. The T1 error rate of each participant was considered the baseline error rate. The pre-stimulation learning rate was calculated by subtracting the T10 error rate from the T1 error rate and dividing by the T1 error rate ([T1 − T10]/T1). The post-stimulation learning rate was calculated by subtracting the T20 error rate from the T11 error rate and dividing by the T11 error rate ([T11 − T20]/T11). The retention rate was calculated by subtracting the R1 error rate from the T20 error rate and dividing by the T20 error rate ([T20 − R1]/T20). The calculated pre-stimulation learning, post-stimulation learning, and retention rates are different from the error rate for each trial, with larger values indicating better learning.

SPSS ver. 25 (IBM Corp., Armonk, NY, United States) was used for all statistical analyses. Analysis of normality using the Shapiro–Wilk test revealed that the baseline error rate of the β-tACS group did not follow normal distribution. Therefore, the Kruskal–Wallis test was used to compare whether participants in each group had equivalent motor abilities. The calculated pre-stimulation learning, post-stimulation learning, and retention rates followed normal distribution across all groups. Mixed analysis of variance (ANOVA) (time factor and stimulus factor) was performed to compare temporal changes in error rates across conditions. Subsequently, one-way ANOVA was used to compare the pre-stimulation learning, post-stimulation learning, and retention rates of each group. The relationships among baseline error, pre-stimulation learning, post-stimulation learning, and retention rates were calculated using Pearson’s product–moment correlation coefficient when data followed normality. Correlation analysis of baseline error rate in the β-tACS group did not follow normality, so Spearman’s rank correlation coefficient was adopted. Furthermore, to compare the calculated correlation coefficients, a test for differences in mother correlation coefficients was performed (Bonferroni correction). The significance level was set at 5% for all analyses.

## 3 Results

### 3.1 Side effect of tACS

Table 1 shows the mean values of side effects assessed during tACS. All 42 participants endured the current for 15 min without interruptions. We believe that sensations related to side effects, such as phosphene and itching, were mild in the γ-tACS and sham groups. In the β-tACS group, side effects were slightly stronger, but not to the extent that the stimulation could be distinguished. No participants reported other side effects during the intervention.

**Table 1:** Mean value of side effects for each tACS condition (mean ± standard deviation).

Phosphene				Itching		
	γ-tACS	β-tACS	sham	γ-tACS	β-tACS	sham
0 min	0	1.50 ± 1.09	0.29 ± 0.61	0.57 ± 0.85	1.36 ± 1.01	0.36 ± 0.50
7.5 min	0.07 ± 0.27	1.21 ± 0.80	0	0.57 ± 0.76	0.64 ± 0.84	0.14 ± 0.36
15 min	0	0.71 ± 0.83	0	0.50 ± 0.76	0.36 ± 0.63	0.07 ± 0.27

0 = “no sensation” to 6 = “very strong sensations.”

### 3.2 Change in error rates over time

**Figure 3:**
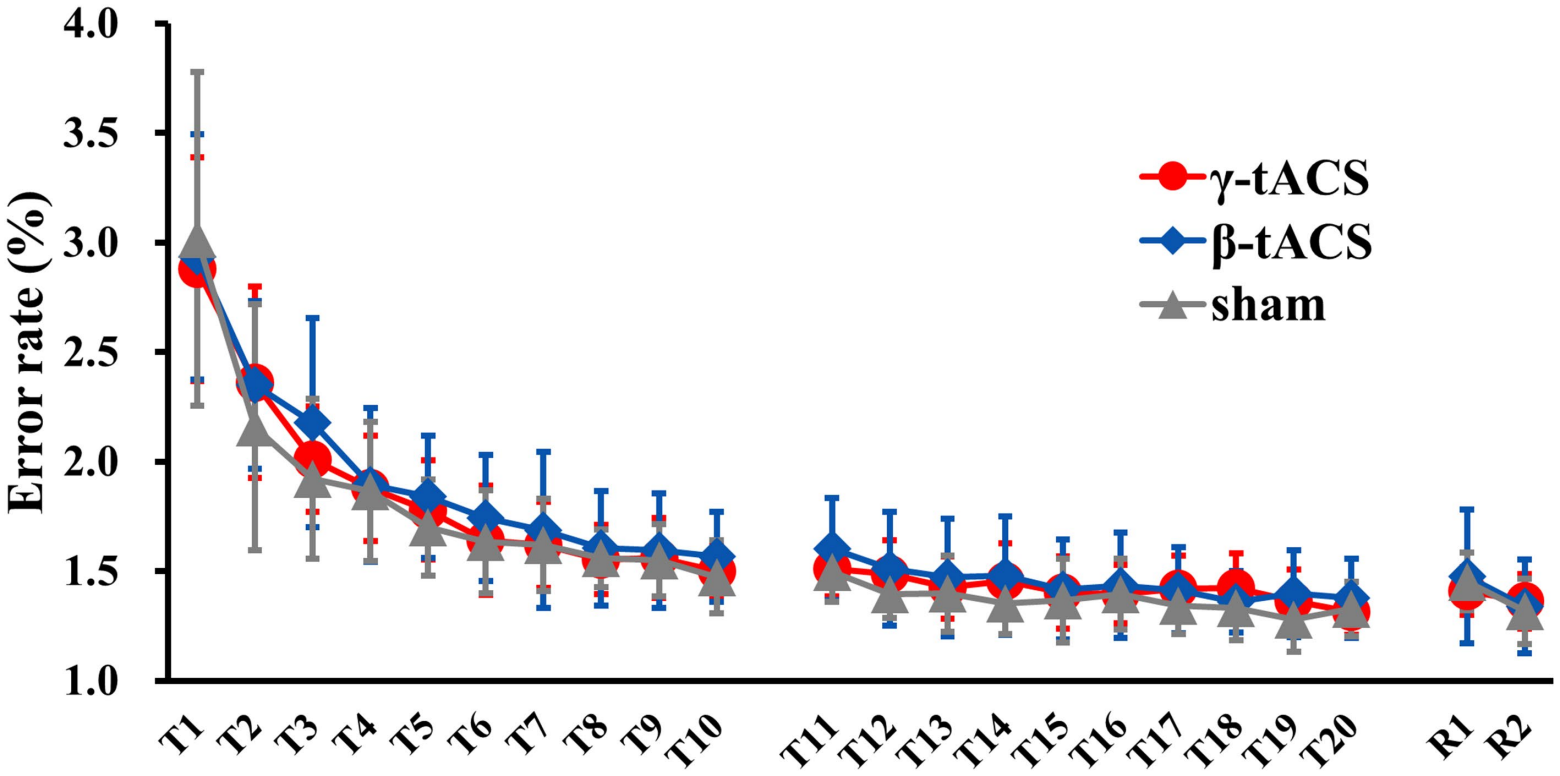
Changes in error rates over time for each group. Solid red, blue, and gray lines show average error rates for the γ-tACS, β-tACS, and sham groups, respectively. Bars indicate standard deviations.

Figure 3 shows the temporal error rate change for each group. No significant differences in baseline error rates were detected between groups (*p* = 0.990). In mixed ANOVA, only the main effect of the time factor (*F*_(2.359, 93.394)_ = 174.307, *p* < 0.001, *η*^2^*p* = 0.817) for the pre-stimulation error rate (T1–T10) was significant; the main effects of the stimulus factor (*F*_(2, 39)_ 409, *p* = 0.667, *η*^2^*p* = 0.021) and interaction (*F*_(4.789, 93.394)_ = 0.925, *p* = 0.466, *η*^2^*p* = 0.045) were not significant. Similarly, for the post-stimulation error rate (T11–T20), only the main effect of the time factor (*F*_(9, 351)_ = 12.554, *p* < 0.001, *η*^2^*p* = 0.244) was significant; the main effects of the stimulus factor (*F*_(2, 39)_ = 1.035, *p* = 0.365, *η*^2^*p* = 0.050) and interaction (*F*_(18, 351)_ = 0.975, *p* = 0.489, *η*^2^*p* = 0.048) were not significant. For the next-day error rate (R1 and R2), only the main effect of the time factor (*F*_(1, 39)_ = 32.598, *p* < 0.001, *η*^2^*p* = 0.455) was significant; the main effect of the stimulus factor (*F*_(2, 39)_ = 0.077, *p* = 0.926, *η*^2^*p* = 0.004) was not significant. No interaction (*F*_(2, 39)_ = 2.590, *p* = 0.088, *η*^2^*p* = 0.117) was observed.

### 3.3 Pre-stimulation learning, post-stimulation learning, and retention rates

**Figure 4:**
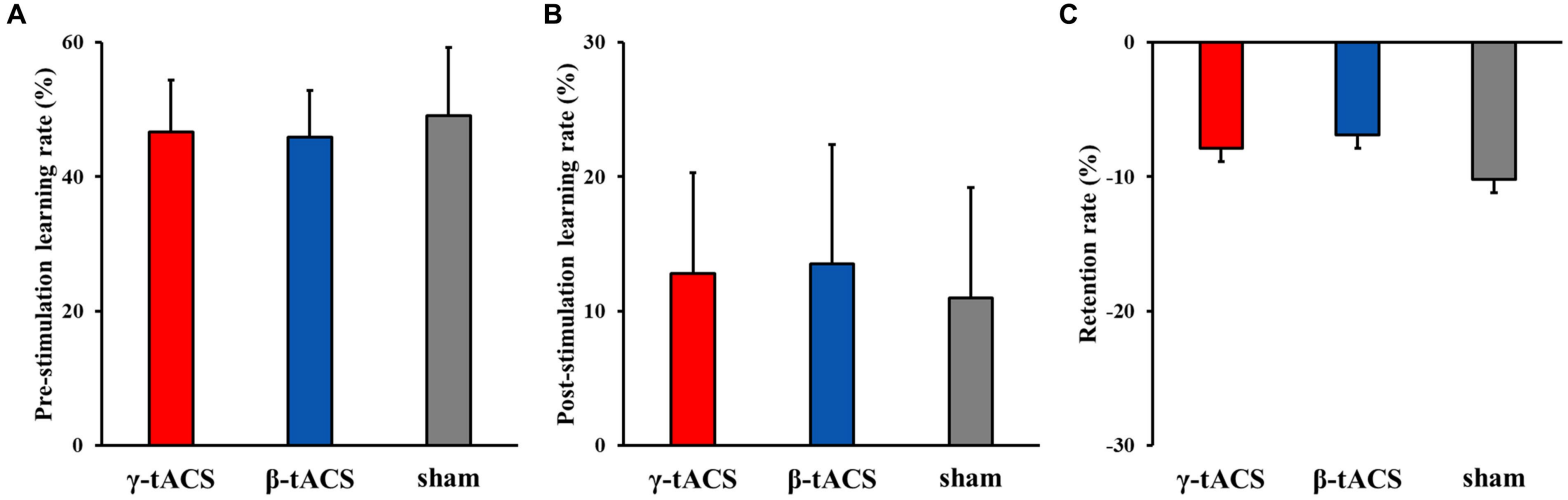
Pre-stimulation learning, post-stimulation learning, and retention rates for each group. Bars indicate standard deviations. **(A)** Pre-stimulation learning rate. **(B)** Post-stimulation learning rate. **(C)** Retention rate.

Figure 4 shows the pre-stimulation learning, post-stimulation learning, and retention rates for each group. No significant differences in pre-stimulation learning (*F*_(2, 39)_ = 0.562, *p* = 0.575, *η*^2^*p* = 0.028; Figure 4A), post-stimulation learning (*F*_(2, 39)_ = 0.333, *p* = 0.719, *η*^2^*p* = 0.017; Figure 4B), and retention (*F*_(2, 39)_ = 0.239, *p* = 0.789, *η*^2^*p* = 0.012) rates (Figure 4C) were detected among the groups.

### 3.4 Correlation between baseline error and pre-stimulation learning rates for each group

Baseline error and pre-stimulation learning rates were positively correlated in all groups (γ-tACS group: *p* < 0.001, *r* = 0.891; β-tACS group: *p* = 0.01, *r* = 0.659; and sham group: *p* < 0.001, *r* = 0.890). Therefore, in all groups, a relationship was observed in which participants with lower baseline performance had higher pre-stimulation learning rates.

### 3.5 Correlation between baseline error and post-stimulation learning rates for each group

**Figure 5:**
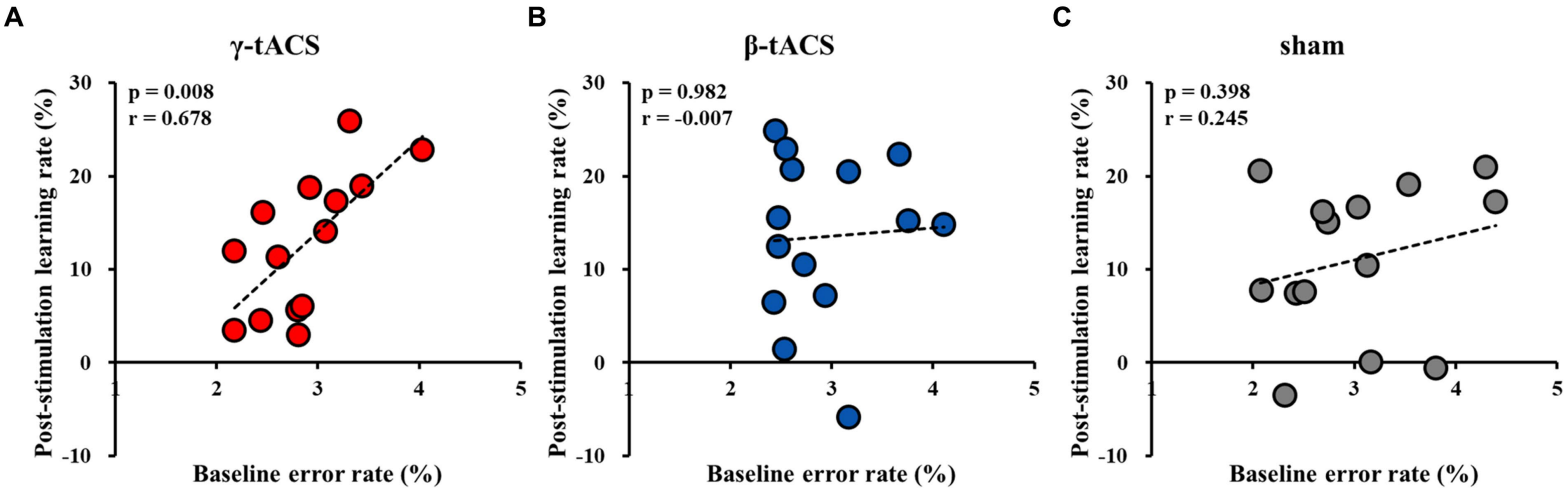
Correlation between baseline error and post-stimulation learning rates for each group. **(A)** γ-tACS group. **(B)** β-tACS group. **(C)** sham group.

Figure 5 shows correlations between baseline error and post-stimulation learning rates for each group. A positive correlation was observed in the γ-tACS group (*p* = 0.008, *r* = 0.678; Figure 5A). However, no significant correlations were observed in the β-tACS and sham groups (β-tACS group: *p* = 0.982, *r* = −0.007, Figure 5B; sham group: *p* = 0.398, *r* = 0.245, Figure 5C). Analysis of differences in mother correlation coefficients revealed no significant difference among the correlation coefficients of each group (γ-tACS group vs. β-tACS group: *p* = 0.076; β-tACS group vs. sham group: *p* = 0.823; and γ-tACS group vs. sham group: *p* = 0.270).

### 3.6 Correlation between pre-stimulation learning and post-stimulation learning rates for each group

**Figure 6:**
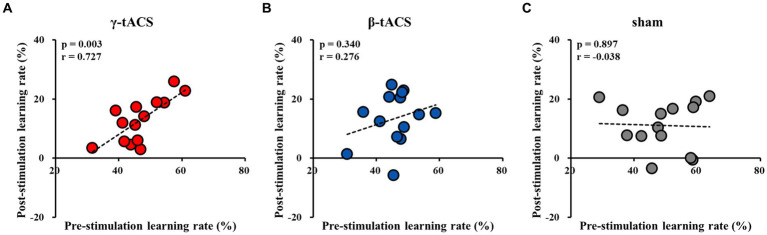
Correlation between pre-stimulation learning and post-stimulation learning rates for each group. **(A)** γ-tACS group. **(B)** β-tACS group. **(C)** sham group.

Figure 6 shows the correlation between pre-stimulation and post-stimulation learning rates for each group. A positive correlation was observed between pre-stimulation and post-stimulation learning rates in the γ-tACS group (*p* = 0.003, *r* = 0.727; Figure 6A). However, no significant correlation was observed between pre-stimulation and post-stimulation learning rates in the β-tACS and sham groups (β-tACS group: *p* = 0.340, *r* = 0.276, Figure 6B; sham group: *p* = 0.897, *r* = −0.038, Figure 6C). Analysis of differences in mother correlation coefficients revealed a significant difference between the correlation coefficients of the γ-tACS and sham groups (*p* = 0.036). However, no significant difference was observed in correlation coefficients between the γ-tACS and β-tACS groups and between the β-tACS and sham groups (γ-tACS group vs. β-tACS group: *p* = 0.204; β-tACS group vs. sham group: *p* = 0.679).

### 3.7 Correlation with retention rate

We investigated the relationship of each rate with the retention rate. There was no significant correlation between baseline error and retention rates in all groups (γ-tACS group: *p* = 0.086, *r* = −0.475; β-tACS group: *p* = 0.113, *r* = −0.443; and sham group: *p* = 0.427, *r* = −0.231). A significant negative correlation was observed between pre-stimulation learning and retention rates only in the γ-tACS group (γ-tACS group: *p* = 0.010, *r* = −0.659; β-tACS group: *p* = 0.509, *r* = −0.193; and sham group: *p* = 0.749, *r* = −0.094). Regarding the relationship between post-stimulation learning and retention rates, no significant correlation was observed in the γ-tACS group, but a significant negative correlation was observed in the β-tACS and sham groups (γ-tACS group: *p* = 0.084, *r* = −0.477; β-tACS group: *p* = 0.017, *r* = −0.624; and sham group: *p* = 0.048, *r* = −0.536).

## 4 Discussion

This study aimed to determine the effects of applying tACS to the SMA on motor learning. Rates of pre-stimulation learning, post-stimulation learning, and retention for a visuomotor tracking task were compared in individuals subjected to different stimulation conditions. No significant differences in pre-stimulation learning, post-stimulation learning, or retention rates were detected among the groups. However, in the γ-tACS group, a positive correlation was observed between baseline error and post-stimulation learning rates and between pre-stimulation learning and post-stimulation learning rates. This suggests that applying γ-tACS to the SMA increases post-stimulation learning rates in individuals exhibiting poor baseline performance and high pre-stimulation learning rates.

The findings of this study suggest that tACS may modulate the oscillatory neural activity of the SMA, potentially influencing its function. tACS is a noninvasive brain stimulation method that modulates brain vibrations in the cerebral cortex and can modulate oscillatory brain activity directly under the electrodes to a specific frequency (Helfrich et al., 2014a,b). Previous studies have demonstrated the modulation of oscillatory neural activity following tACS (Neuling et al., 2013; Helfrich et al., 2014a). Our previous study showed that individuals exhibiting lower baseline performance in a bimanual motor task demonstrated improved motor abilities with γ-tACS targeting the SMA, whereas those exhibiting higher baseline performance showed improvement with β-tACS (Miyaguchi et al., 2020a). In the present study, a correlation with baseline performance was observed in the γ-tACS group but not in the β-tACS and sham groups. Therefore, the relationships observed in this study partially support our previous findings. A plausible explanation for the present study results is that γ-tACS applied to the SMA facilitated the updating of motor plans in participants exhibiting low baseline performance and high pre-stimulation learning rates. Mary et al. (2017) reported that young healthy individuals exhibiting higher learning performance demonstrated a strengthened neural network between the SMA and the primary sensorimotor cortex (SM1), whereas those exhibiting lower learning performance demonstrated a strengthened neural network between the cerebellum and SM1. Thus, SMA activity may be necessary for motor tasks in individuals exhibiting high pre-stimulation learning rates but not for those exhibiting low pre-stimulation learning rates. Furthermore, γ-band activity in the SMA is involved in the updating of motor plans (Hosaka et al., 2016). Therefore, it is possible that in participants exhibiting high pre-stimulation learning rates in this study, SMA γ-band activity updated motor plans during repetitive movement practice. In participants exhibiting low learning rates, the neural network between the cerebellum and SM1 is more involved than the SMA (Mary et al., 2017), resulting in low demand for SMA activity. Thus, γ-tACS may not affect post-stimulation learning rates in these individuals. In contrast, in the β-tACS group, it is conceivable that γ-oscillatory activity was disturbed in participants exhibiting lower baseline performance and higher pre-stimulation learning rates. The β-oscillatory activity of the SMA is involved in maintaining motor planning (Hosaka et al., 2016). It is possible that the correlation observed in the γ-tACS group was not found in the β-tACS group because the application of β-tACS to the SMA did not affect the updating of motor plans during exercise practice. This was also true for the sham group, suggesting that no correlation could be established because updating of the motor plan could not be promoted. Furthermore, in this study, a relationship was observed across all groups where participants exhibiting lower baseline performance showed higher learning rates before stimulation. This suggests that participants exhibiting poor baseline performance have higher pre-stimulation learning rates because they have more room for improvement in motor skills. Nevertheless, only the γ-tACS group showed a correlation with post-stimulation learning rates. Therefore, the effect of increasing post-stimulation learning rates in participants exhibiting low baseline performance and high pre-stimulation learning rates may be specific to γ-tACS. However, in this study, no significant difference was observed in the post-stimulation learning rate between each group. This suggests that the effect of applying γ-tACS to the SMA on motor learning may not be effective for all participants but may vary depending on the individual’s motor ability.

Additionally, administration of tACS to the SMA did not affect the next-day retention rate. Therefore, although the stimulation effect in this study improved post-stimulation learning in participants exhibiting low baseline performance and high pre-stimulation learning rates, it did not extend to next-day motor skills. Steele and Penhune (2010) observed activity in areas such as the SMA during the early stages of learning, but activity decreased as learning progressed, suggesting that motor planning activity may have decreased. In the present study, it is possible that next-day SMA activity decreased with repeated exercise practice and updating of the exercise plan. Thus, the effect of applying tACS to the SMA may not have carried over to the next day.

This study has several limitations. First, participants were not asked at the end of the experiment whether the stimulus they received was real or sham. Therefore, their blinding to the stimuli may not have been optimal. Second, brain activity was not measured. Therefore, it is unclear whether oscillatory brain activity was altered by applying tACS to the SMA. Sugata et al. (2018) reported that oscillatory brain activity in the β-band increased after applying γ-tACS to M1. Consistently, in the present study, γ-tACS may have increased oscillatory activity in frequency bands other than the γ-band. Moreover, this study did not investigate the electric field through electric field simulation. Therefore, it remains unclear whether the stimulation parameters employed effectively modulated brain activity in the SMA. Furthermore, because head size varied between participants, it is necessary to use an electroencephalogram cap and optimize electrode placement through simulation. By conducting electric field simulations, it may be possible to enhance focus and effectively facilitate motor learning. Finally, the study had 14 participants in each group, potentially leading to small sample sizes and substantial variability in tACS efficacy. Recognizing this sample size constraint and increasing the number of participants in future studies may yield more reliable results.

There are several other issues that must be addressed in the future. First, this study was unable to examine the effects of repeated interventions. Thus, further investigation is required to determine whether similar effects can be obtained when tACS is repeated over several days. In the future, investigating the effects of intervention over the next few days will likely contribute to a deeper understanding of the stimulation effect. Second, it is necessary to consider changing the timing of tACS intervention. Review papers reporting the effects of tACS on athletic performance suggest that online tACS can effectively improve athletic performance (Hu et al., 2022). Although this study focused on the effects on motor learning after stimulation, tACS intervention during motor practice may more effectively promote motor learning. Despite these limitations and challenges, few reports have described stimulation methods that enhance post-stimulation learning of motor skills. To the best of our knowledge, this study is the first to demonstrate the effect of applying tACS to the SMA on motor learning. Our findings provide a basis for clarifying the effects of applying tACS to the SMA on motor learning. In the future, by addressing these limitations and issues, it may be possible to utilize tACS to contribute to the development of new motor learning programs tailored to individual motor abilities.

## 5 Conclusion

This study demonstrates that γ-tACS applied to the SMA improved post-stimulation learning rates in participants with lower baseline performance and higher pre-stimulation learning rates. We attribute this outcome to the updating of motor plans by applying γ-tACS to the SMA. Hence, applying γ-tACS to the SMA based on an individual’s motor and learning abilities may effectively promote motor learning.

## Data availability statement

The raw data supporting the conclusions of this article will be made available by the authors, without undue reservation.

## Ethics statement

The studies involving humans were approved by Ethics Committee of Niigata University of Health and Welfare, Niigata, Japan. The studies were conducted in accordance with the local legislation and institutional requirements. The participants provided their written informed consent to participate in this study.

## Author contributions

SY: Conceptualization, Data curation, Investigation, Validation, Visualization, Writing – original draft. SM: Conceptualization, Formal analysis, Funding acquisition, Methodology, Project administration, Resources, Supervision, Writing – original draft, Writing – review & editing. TO: Conceptualization, Investigation, Validation, Writing – original draft. YI: Project administration, Supervision, Writing – review & editing. NO: Project administration, Supervision, Writing – review & editing. HO: Funding acquisition, Project administration, Resources, Supervision, Writing – review & editing.
